# Endpoint surrogacy in oncology Phase 3 randomised controlled trials

**DOI:** 10.1038/s41416-020-0896-5

**Published:** 2020-05-26

**Authors:** Jianrong Zhang, Meagan R. Pilar, Xiaofei Wang, Jingxia Liu, Herbert Pang, Ross C. Brownson, Graham A. Colditz, Wenhua Liang, Jianxing He

**Affiliations:** 1 Department of General Practice, Melbourne Medical School; Cancer in Primary Care Research Group, Primary Care Collaborative Cancer Clinical Trials Group (PC4), Centre for Cancer Research, Faculty of Medicine, Dentistry and Health Sciences, University of Melbourne; Victorian Comprehensive Cancer Centre, Melbourne, VIC Australia; 2 Prevention Research Center, Brown School at Washington University in St. Louis, St. Louis, MO USA; 3 Department of Biostatistics and Bioinformatics, Duke University School of Medicine, Durham, NC USA; 4 Department of Surgery, Division of Public Health Sciences, Washington University School of Medicine, St. Louis, MO USA; 5 School of Public Health, Li Ka Shing Faculty of Medicine, University of Hong Kong, Hong Kong, China; 6 Alvin J. Siteman Cancer Center, St Louis, MO USA; 7 Department of Thoracic Surgery and Oncology, The First Affiliated Hospital of Guangzhou Medical University, State Key Laboratory of Respiratory Disease, National Clinical Research Center for Respiratory Disease, Guangzhou Institute of Respiratory Disease, Guangzhou, Guangdong China

**Keywords:** Randomized controlled trials, Cancer epidemiology

## Abstract

Endpoint surrogacy is an important concept in oncology trials. Using a surrogate endpoint like progression-free survival as the primary endpoint—instead of overall survival—would lead to a potential faster drug approval and therefore more cancer patients with an earlier opportunity to receive the newly approved drugs.

## Main

Randomised controlled trial (RCT) is a highly rigorous study design. In oncology, it determines whether a new cancer drug or a new indication of an existing cancer drug has better clinical benefit compared with the standard of care or placebo, and offers the empirical evidence for a regulation authority like the European Medicines Agency or the US Food and Drug Administration (FDA) to approve the new drug or the new indication.^1^

In RCTs, selecting valid endpoints that capture clinical benefit is very important. For measuring efficacy, overall survival (OS) and progression-free survival (PFS) are the most common endpoints, defined as “the time from randomisation until death from any cause” and “the time from randomisation until objective tumour progression or death”, respectively.^2^ In Phase 3 trials, OS is the standard endpoint to establish clinical benefit; however, it requires a longer period of follow-up, often relatively larger number of events, and therefore a higher cost compared with other endpoints like PFS.

To address these limitations of OS, the concept of surrogate endpoint was born. According to the US Federal Food, Drug and Cosmetic Act, the term “surrogate endpoint” means a marker (e.g. laboratory measurement, radiographic image) that “is not itself a direct measurement of clinical benefit, and—(A) is known to predict clinical benefit and could be used to support traditional approval of a drug or biological product; or (B) is reasonably likely to predict clinical benefit and could be used to support the accelerated approval of a drug or biological product”. For example, PFS could be the surrogate endpoint to predict the result of OS in Phase 3 trials.^2^ With a shorter period of follow-up, often smaller number of events, and therefore a trial with less cost, using a surrogate endpoint as the primary endpoint—instead of OS—would lead to a potential faster drug approval and therefore more cancer patients with an earlier opportunity to receive the newly approved drugs.^3^

Prior to the use of a surrogate endpoint in clinical trials to establish the efficacy of the new drugs, a question to be answered is: how can we know how accurate the prediction is? Conducting validation studies is an answer. To date, numerous validation studies have been conducted. Recently in the *British Journal of Cancer*, Belin et al.^4^ published a complete overview of validation studies on PFS for OS, summarising the validity of PFS as a surrogate endpoint for OS, and describing the characteristics of the methodologies used in those validation studies. Two main findings have been reported by the authors. First, only half (52%) of all 91 validation studies (involving 24 cancer localisations) concluded on the validity of PFS for OS; of those studies, only half (51%; 24 studies) indicated a good endpoint surrogacy (criteria used in this review: trial-level *R*^2^ ≥ 0.6). Second, most of those validation studies utilised a meta-analytic approach per recommended, but a remarkable heterogeneity in methods and reporting was noticed. Specifically, the methods were heterogeneous in the evaluation at patient or trial level, and in the trial-level evaluation on the aggregate measures based on one arm (median PFS at a clinically meaningful timepoint), or the treatment effects based on two arms (hazard ratio, difference of median, ratio of median).^4^

These findings have profound implication to design future clinical trials. The first main finding emphasises that endpoint surrogacy of PFS for OS is not universally valid, given that all included validation studies were conducted based on different interventions and different indications (e.g. treatment regimen, treatment line, cancer type, cancer stage). For example, there is currently a lack of strong evidence supporting the validity of PFS for OS in immunotherapies.^4,5^ Therefore, we encourage future validation studies that should have focus(es) on specific intervention and specific indication. Through such an approach, one can assess how valid a surrogate endpoint is to predict clinical benefit according to each specific intervention and specific indication. To better accomplish validation studies for specific intervention and specific indication, we need a large pool of clinical trials data in which both OS and the surrogate endpoints were captured. An easy access to the data from completed clinical trials from many pharmaceutical industry or government spooned trials for research use is the key to such success. We also encourage conducting more validation studies focusing on not only PFS, the most common surrogate endpoint for OS, but also other potential endpoints like metastasis-free survival^6^ and milestone survival.^7^ Furthermore, these potential surrogate endpoints have focus(es) on specific intervention and specific indication.

Corresponding to the second main finding, we agree with Belin et al.^4^ that developing and applying recommendations on the methodology and reporting of validation analyses is important. Related recommendations on reporting have been well established in the ReSEEM guidelines,^8^ which provide useful suggestions on methodology to improve consistency in validation studies. Consensus is still needed with regard to more nuanced aspects of methodology, especially at the policy level. For example, there is no consensus on the extent to the prediction indicating a valid endpoint surrogacy (e.g. trial-level or patient-level *R*^2^? *R*^2^ ≥ 0.?). Also, no consensus is made on the validation evaluation that whether and how an adjustment by trial characteristics should be applied, and that whether the aggregate measures or the treatment effects should be used. With respect to which aggregate measures or treatment effects should be used, however, it may be too early to develop a universal recommendation, given the current debate between the use of traditional aggregate measures (e.g. median survival) or treatment effects (e.g. hazard ratio) compared with the aggregate measures or treatment effects based on newer endpoints (e.g. milestone survival^7^) and/or newer statistical methods using the landmark analysis^9^ or restricted mean survival time.^10^ Therefore, we suggest current validation studies to consider any possible aggregated measures or treatment effects in their evaluation of validity.

## Data Availability

Not applicable.
